# Measuring tissue water potential in marine macroalgae via an updated Chardakov method

**DOI:** 10.1093/aobpla/plad055

**Published:** 2023-08-22

**Authors:** V L Gibson, A Richards Donà, C M Smith

**Affiliations:** School of Life Sciences, University of Hawai‘i at Mānoa, 310 Maile Way, St John 101, Honolulu, HI 96822, USA; Water Resources Research Center, University of Hawai‘i at Mānoa, 2540 Dole Street, Holmes Hall 293, Honolulu, HI 96822, USA; Hawaiʻi Institute of Marine Biology, University of Hawaiʻi at Mānoa, 46-007 Lilipuna Road, Kāneʻohe, HI 96744, USA; School of Life Sciences, University of Hawai‘i at Mānoa, 310 Maile Way, St John 101, Honolulu, HI 96822, USA; Water Resources Research Center, University of Hawai‘i at Mānoa, 2540 Dole Street, Holmes Hall 293, Honolulu, HI 96822, USA; School of Life Sciences, University of Hawai‘i at Mānoa, 310 Maile Way, St John 101, Honolulu, HI 96822, USA

**Keywords:** water relations, osmotic response, salinity tolerance, phycology

## Abstract

Regulation of tissue water potential is a key mechanism in macroalgal osmotic responses to changing external osmotic conditions, which are common in tidally influenced estuarine and intertidal systems. Nevertheless, significant knowledge gaps exist in our understanding of osmotic responses in macroalgae because few methods measure osmotic potential within macroalgal tissues. Leaf psychrometers have furthered understanding of osmotic potentials in terrestrial plant water relations, yet these have not been developed to measure the range of highly negative potential values found in marine macroalgae. To address these gaps, we present an effective, updated version of the Chardakov method to measure tissue water potential in macroalgae. Here, we present a case study examining macroalgal response in tissue water potential by two morphologically and evolutionarily distinct species, *Ulva lactuca* (Chlorophyta) and *Hypnea musciformis* (Rhodophyta) to four paired salinity and nutrient treatments at two temperatures. These treatments simulate a gradient from full coastal ocean conditions to brackish submarine groundwater discharge, an ecosystem type found on basaltic shorelines. Both algae demonstrated plasticity in osmotic response to submarine groundwater discharge with significant positive correlations between tissue water potential and proportion of submarine groundwater discharge in the treatment. These results are the first to describe macroalgal response in tissue water potential, a first step to understanding algal physiological ecology in such complex coastal environments. This revised Chardakov method is a valuable tool to better understand species-specific osmotic responses to ecologically relevant conditions, and can augment the study of other tidal systems and ontogenetic stages.

## Teaser

Marine plants occurring in tidally-influenced estuarine nearshore ecosystems experience daily oscillations from fresh to marine salinities. Under the stress of changing external salinities regulation of solute concentrations within plant cells is critical to continued growth and photosynthesis. However, a lack of available methodologies has prevented significant breakthroughs in understanding of water relations in marine plants since the 1980s. This work updates the Chardakov Method, a classic method of measuring tissue water potential. This simple and cost-effective method is applied to examining the regulation of internal cellular solute concentrations for two Hawaiian marine plant species under simulated conditions of nearshore lowered salinity and higher nutrients. This work demonstrates the use of this methodology for measuring marine plant water potentials, and measures salinity-driven regulation of internal cellular solute concentrations for two species. The authors encourage the use of this method for further investigation of water relations in marine plants under oscillating salinities.

## Introduction

Salinity tolerance plays a key role in determining the distribution of macroalgae (Biebl 1952; Bird *et al.* 1979; Bird and McLachlan 1986; Larsen and Sand-Jensen 2006), and osmotic responses are likely key mechanisms underlying algal performance, particularly in systems with highly variable conditions of salinity common in intertidal, estuarine and nearshore ecosystems influenced by submarine groundwater discharge (SGD; Kirst 1989; Dulai *et al.* 2021). Submarine groundwater discharge is a cryptic yet common feature on basaltic coastlines, which delivers a tidally driven diurnal pulse of fresh to brackish, nutrient-rich basal groundwater to the nearshore ecosystem (Burnett *et al.* 2003; Moore 2010; Beusen *et al.* 2013; Amato *et al.* 2016). Osmotic responses to changes in external osmotic conditions are poorly understood in macroalgae (Kirst 1989) but likely influence macroalgal distribution dynamics. Mechanisms that govern salinity tolerance in terrestrial plants are well established (Munns and Tester 2008). Multiple mechanisms for salinity tolerance in macroalgae have been described (Blinks 1949; Kirst 1989), and other macroalgal responses to salinity stress have been measured (Nejrup and Pedersen 2012; Pereira *et al.* 2017; Amato *et al.* 2018; Solami 2020). However, the inability to measure osmotic potentials within macroalgal tissues has limited developments in understanding osmotic response in tissue water potential (TWP) regulation directly (Kirst 1989; Jain *et al.* 2021). The processes associated with osmotic adjustment in response to changing salinity have instead been inferred from ion and organic osmolyte concentrations (Hastings and Gutknecht 1976; Kirst 1977; Reed *et al.* 1981) or examined as the effects of osmotic stress on growth (Binbin and Dinghui 2015; Samanta *et al.* 2019), fine structure (Reed *et al.* 1980; Pereira *et al.* 2017; Dulai *et al.* 2021), photosynthesis and respiration (Dawes *et al.* 1978; Kirst 1989), and pigment concentrations (Solami 2020). Few studies have directly measured macroalgal TWP (Bisson and Gutknecht 1977; Reed 1983; Kawamitsu *et al.* 2000), limiting a comprehensive understanding of plasticity in response to changing salinity conditions needed to project the effects of anthropogenic and climate change impacts on nearshore salinity regimes of reef ecosystems (Dulai *et al.* 2021). Here, an updated Chardakov method (Chardakov 1948; Knipling 1967; O’Leary 1970) is employed to demonstrate its use in examining macroalgal TWP response to simulated conditions of salinity and nutrients across a gradient from oceanic to SGD-influenced nearshore reef conditions, and to two temperature treatments.

When compared with freshwater macroalgae, marine macroalgae with their TWP adjusted to marine conditions contain higher solute concentrations, thus exhibiting more negative TWP values to match the water potential of the marine bathing medium (Kirst 1977, 1989; Zimmermann 1978; Kirst and Bisson 1979). If exposed to hypo-osmotic conditions without TWP regulation, these marine-adjusted cells experience an osmotic influx that could lead to cell lysis (Zimmermann 1978; Kirst 1989). Thus, when exposed to hypo-osmotic conditions, macroalgae are expected to lower internal solute concentrations to mirror external solute concentrations (Kirst 1977, 1989; Zimmermann 1978; Kirst and Bisson 1979). This response yields less negative TWP, reduces the potential gradient and decreases the net movement of water into the plant cell, thereby avoiding cell lysis (Zimmermann 1978; Kirst 1989). Tissue water potential regulation is achieved through physiological manipulation of cellular ion and organic osmolyte content via response pathways that appear to vary within and across phylogenies (Zimmermann 1978; Kirst and Bisson 1979; Kirst 1989; Koide *et al.* 1989). We predicted that these responses occur within macroalgae experiencing SGD, as SGD introduces a tidally driven flux of fresh to brackish water with lower salinity and cooler temperatures along with elevated nutrients from the basal aquifer to the nearshore ecosystem (Johnson *et al.* 2008; Knee *et al.* 2010; Bishop *et al.* 2017).

Submarine groundwater discharge is important in coastal ecosystems because it drives productivity (Kløve *et al.* 2014; Rohde *et al.* 2017) and exposes benthic macroalgae to tidally driven oscillations in temperature, salinity and nutrient availability (Dulai *et al.* 2021). To examine macroalgal osmotic response to SGD conditions on Hawaiʻi’s reefs, TWP was measured for two species, *Hypnea musciformis* (Rhodophyta) and *Ulva lactuca* (Chlorophyta), incubated under four laboratory-simulated conditions representing a gradient from SGD conditions of low salinity and elevated nutrient conditions to ambient nearshore salinity of 35 ppt and low nutrients (following Johnson *et al.* 2008; Amato *et al.* 2018). The four salinity/nutrient treatments were run at two temperatures (20 and 27 °C), representative of the range found at an SGD-influenced site, Waiʻalae ʻIki, Oʻahu (Dulai *et al.*, 2021).

The Chardakov method is a conceptually simple and reproducible technique for measuring plant TWP (Chardakov 1948; Knipling 1967). Water potential, or Ψ_w_, is the measure of the energy state of water, and is calculated as the sum of the pressure potential, Ψ_p_, the solute potential, Ψ_s_ and the gravity potential, Ψ_g_, of the solution:


$$\Psi_{\text{w}}=\Psi_{\text{p}}+\Psi_{\text{s}}+\Psi_{\text{g}}$$ (1)

Tissue water potential is the Ψ_w_ within a plant or animal tissue (Koide *et al.* 1989; Taiz *et al.* 2014). This method was initially developed to measure ‘suction pressure’ of plants to infer the implications of soil moisture in cotton farming by the USSR in Tajikistan (Chardakov 1948, 1953, 1955), and was later published in English (Knipling 1967; Soule 1968). While some published studies have demonstrated the use of the Chardakov method for measuring TWP for terrestrial plants (Knipling 1967; Knipling and Kramer 1967; Yuda and Okamoto 1967; O’Leary 1970; Debergh *et al.* 1981; Matsuda and Riazi 1981; Robertson *et al.* 1985; Gorton 1987; Chapotin *et al.* 2006), no published studies demonstrate the use of this method for macroalgae. Rather, macroalgal studies have measured TWP using freeze point depression measurements (Bisson and Gutknecht 1977), psychrometry (Reed 1983; Kawamitsu *et al.* 2000) or inferred changes by measuring concentrations of inorganic ions and organic osmolytes (Blinks 1949; Dickson *et al.* 1982; Kirst 1989). Similarly, osmoregulation in fish has been inferred from measurements of inorganic ions and organic osmolytes (Moorman *et al.* 2015). A modified Chardakov method has, however, been used for measuring the osmotic characteristics of anuran blood and other bodily fluids (Soule 1968). Until recent developments in nanotechnology (Jain *et al.* 2021), Chardakov’s method was the only established way to measure TWP within living biological systems (Soule 1968).

This method measures TWP by incubating fragments of a tissue sample in a range of osmotic solutions of known molar concentrations (Knipling 1967; Devlin and Witham 1983). By adding a miniscule amount of a dye to each solution of the experimental system, the concentration of solute within the plant cells is matched to unaltered test osmotic solutions of the same molality (Knipling 1967; Devlin and Witham 1983). The post-incubation density of the incubation solution is measured, and a match with the test solution is found, by inserting dyed droplets of the incubation solution into unaltered test solutions of the same concentration (Knipling 1967; Devlin and Witham 1983). This method lends itself particularly well to field and laboratory measurements of TWP in macroalgae as their simple morphologies and tissue types allow potential-driven osmosis to occur freely across the cellular membrane. Because of their lack of cuticle, protective barriers, trichomes or hairs at the surface interface with the surrounding bathing medium, macroalgae are not subject to complications described for the use of Chardakov with terrestrial plants such as sap leakage and suppression of water exchange caused by waxy cuticles (Knipling and Kramer 1967).

Three other methods of measuring TWP that could be applied include tissue volume method, pressure chamber techniques and psychrometry (Knipling and Kramer 1967; Koide *et al.* 1989; Gekas 2001). However, these methods are slow and destructive to the measured tissue, the tissue microenvironment, or both (Jain *et al.* 2021). Recently developed hydrogel nanoreporters are minimally destructive to tissues as these respond to changes in surrounding potential by swelling, changing the emission spectrum for dye molecules within the nanoreporters and allowing for real-time TWP measurement via fibre optics (Jain *et al.* 2021). These breakthroughs provide an opportunity for significant developments in understanding osmotic responses in all organisms. Nevertheless, the economic costs, equipment and skill sets required for the use of nanoreporters remain prohibitive for most users, and nanoreporter methods have not yet been established for macroalgae.

Here, we present an updated version of Chardakov’s method as a robust measure of TWP in marine plants with minimal tissue death, maximal relevance in field or laboratory conditions, and low costs per run. This method can be implemented by users with minimal training. We suggest the use of this method to address the large gaps that remain in understanding osmotic responses by macroalgae.

## Methods

### Chardakov method summary

**Figure 1.**
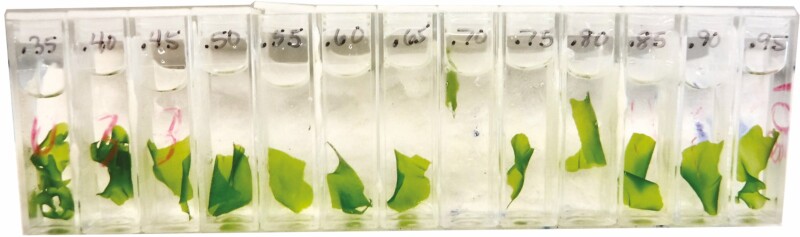
Fragments of *U. lactuca* under incubation in an updated Chardakov incubation array. The incubation array is labelled with the molar sorbitol concentrations of each incubation liquid, from 0.35 to 0.95 M with increasing increments of 0.05 M.

**Figure 2.**
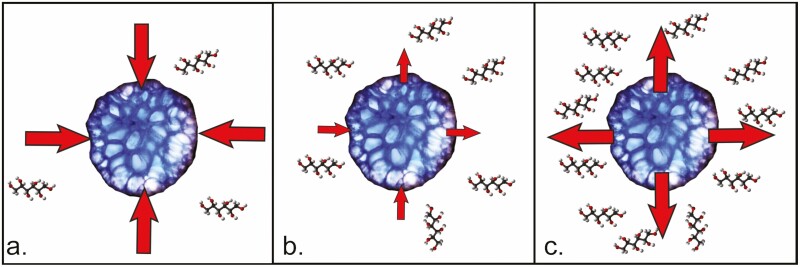
(A) A cross-section of *H. musciformis* represents a plant in 0.35 M sorbitol bathing medium, where Ψ_cell_ > Ψ_solution_ and the potential gradient drives water into the plant cells. (B) A cross-section of *H. musciformis* represents a plant in 0.60 M sorbitol bathing medium, where Ψ_cell_ = Ψ_solution_ and no net movement of water occurs. (C) A cross-section of *H. musciformis* represents a plant in 0.95 M sorbitol bathing medium, where Ψ_cell_ < Ψ_solution_ and the potential gradient drives water out of the plant cells.

**Figure 3.**
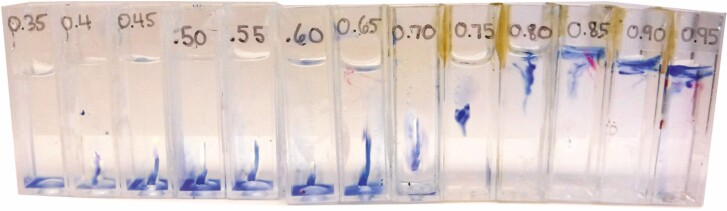
Blue colored test droplets from the incubation solution within the modified Chardakov test array. Here we get a reading of 0.75 M where the droplets transition from sinking in 0.35-0.70 M to floating in 0.8-0.95, with a “hover” seen at 0.75 M, indicating no change in osmotic potential of the incubation solution. Note that the user must observe and record the behavior of each droplet (float, sink, or hover) immediately after insertion into the test solution, to avoid artifacts.

In the Chardakov method, TWP is measured by incubating replicate fragments of tissues from a single sample in a series of graded, small-volume solutions (i.e. sorbitol) of known molar concentration (see Fig. 1, **Supporting Information—Fig.**). One tissue fragment is incubated in each solution, and osmosis is allowed to occur within each container, such that the potential difference-driven osmotic gradient between the plant cells, Ψ_cell_, and the surrounding solution, Ψ_solution_, drives water in or out of the plant fragment, thus altering the molar concentration of the incubation solution. When Ψ_cell_ > Ψ_solution_ water is driven into the plant cells and the incubation solution becomes ‘heavier’ or more concentrated (Fig. 2A). When Ψ_cell_ < Ψ_solution_, water moves out of plant cells, and the incubation solution becomes ‘lighter’ or less concentrated (Fig. 2C). Where Ψ_cell_ = Ψ_solution_ no net change in the bathing medium occurs as there is no gradient to drive osmosis (Fig. 2B). Inserting droplets of dyed incubation solution into unaltered test solutions of the same initial molar concentration allows the user to visually determine if changes in the molar concentration of the bathing medium have occurred. The user visually determines if the droplet sinks (incubation solution is heavier, indicating Ψ_cell_ > Ψ_solution_), hovers (incubation solution has no net change, indicating Ψ_cell_ = Ψ_solution_), or floats (incubation solution is lighter, indicating Ψ_cell_ < Ψ_solution_; see test droplets in Fig. 3 and illustrated steps in **Supporting Information—Figs S1–S4**). The molar concentration where the incubation droplet hovers since Ψ_cell_ = Ψ_solution_ may then be recorded for the TWP and converted to MPa **[see****Supporting Information—2.1** for conversion**]**.

#### Preparation

Prior to day-of measurement activities, sorbitol solutions were mixed, Chardakov arrays were constructed, and arrays were filled with the appropriate sorbitol solution over a range of 0.35–0.95 M in increments of 0.05 M. Each measurement used two complete arrays containing 13 cuvettes filled with 4.5 mL of the appropriate sorbitol concentration.

#### Sorbitol mixing and storage

Sorbitol solutions were mixed using High Purity D Sorbitol (HOH_2_C(CH(OH))_4_CH_2_OH) and distilled water from the St John Plant Science Building still (VaPure VCS-25, Mueller BIOPHARM Systems, Springfield, Ml). Sorbitol was weighed using a mass balance with ±0.001 g (Mettler PE 360 Delta Range) and mixed with distilled water using a 1000 mL volumetric flask and a Nuova combined heat and magnetic stir bar mixer. Clean glassware was used throughout to avoid contamination of solutions. Once mixed, sorbitol solutions were refrigerated at 15 °C. For this study, sorbitol solutions were mixed within 4 weeks of analyses.

#### Chardakov array preparation

Arrays of cuvettes for Chardakov tests were constructed by gluing linear arrays of 13 polystyrene cuvettes with epoxy glue. The cuvettes allowed for easy incubation of plant parts and clear visual assessment of results. These advantages represent significant improvements in the ease of using this method from those previously described, which used individual round glass vials. Further, these Chardakov arrays have a more accurate flat view plane than round vials, take less time to organize and use, are stable in the field and consume less sorbitol solution per measurement. Cuvette lids were used to minimize evaporation or spillage.

Individual cuvettes within the array were labelled 0.35 to 0.95 M, increasing in 0.05 M increments from low to high concentration in ascending order (left to right; Fig. 3). Prior to the TWP tests, cuvettes were filled with 4.5 mL of the sorbitol solution matching to their label using a BD 60 mL graduated syringe with a 20 Ga blunt needle tip, and lids were secured. The graduated syringe was rinsed three times with distilled water and dried with a clean paper towel between concentrations, or replaced as needed. Once filled, the arrays were arranged upright in plastic storage boxes and stored at 20 °C until the experiment.

#### Day-of experiment preparation

Chardakov arrays were refrigerated until approximately 1 h before use, when the arrays were placed on a shaker table to homogenize the sorbitol solutions and were allowed to warm to ambient temperature. This step avoids additional viscosity and variable or cold temperatures. Each test array was handled along with its paired incubation array to avoid differences at the time of measurement.

### Chardakov measurements of macroalgal TWP

#### Incubation

For each analysis, a single algal specimen was patted dry with paper towels until no surface moisture was visible, then cut into 13 fragments of similar size, set for each species. Fragments were cut to length using a razor blade and a standard ruler with millimetre divisions. To standardize fragments for *U. lactuca*, 2 cm^2^ fragments were cut from vegetative blade tissue; specialized tissues such as holdfasts and reproductive tissues were avoided. To standardize fragments for *H. musciformis*, 2 cm long sections of the main axis were selected from near the tips of plants, with two to three branchlets of 2 cm length or less. Parasites, bleached tissues and holdfasts were avoided. Fragments were then photographed.

Once algal fragments for a single specimen were prepared, the incubation and test array were prepared and mixed with lids for 5 s, to ensure homogeneity within cuvettes. For each cuvette in the incubation array, one tissue fragment was inserted, completely submerged in the incubation liquid and the lid replaced (Fig. 2). Once all fragments were inserted and lids secured, the array was again mixed for 5 s by hand and then inserted in an upright position into a holding box for incubation. For control purposes, the test array was also mixed for 5 s by hand and inserted into the holding box. Samples were then incubated for 30–60 min (*n* = 34 samples). Due to limited manpower and the volume of samples processed in one day, in this experiment it was necessary to incubate a majority of samples for 60–120 min (*n* = 111), though in extreme cases samples were incubated for 120–133 min (*n* = 8). We took this opportunity to continue measurements so that we could later analyse statistically if these differing incubation times impacted the result. Varying incubation times are difficult to avoid during staggered measurements as the time taken to implement and read each measurement can vary from 3 to 10 min. Incubation times of over an hour but less than 3 h did not impact the results in this and previous studies and were accounted for in our statistical analyses.

#### Implementing and data gathering from the Chardakov analyses

Following incubation, the incubation array and the corresponding test array were held in a white plastic tray with two compartments to create a flat surface and a white backdrop. For control during this experiment, each incubation array with algal fragments was photographed, and the temperature of the 0.35 M sorbitol incubation and the test solution were taken for a subset of the measurements (*n* = 112). Temperature differences >2 °C required waiting for the solutions to reach similar temperatures. In some cases (*n* = 12), it was not possible to wait for vials to reach similar temperatures while staying within the incubation time frame, thus some samples were analysed with larger temperature differences (2.5–7 °C). No significant effect of these differences was seen in statistical analysis of the data and thus we did not omit these data. For visual analysis, two to four crystals of water-soluble Aniline Blue dye (Merck & Co.) were dropped into each incubation cuvette **[see****Supporting Information—Fig. S2****]**. Lids were replaced, the array was shaken three times and the dye was allowed to mix with the incubation solution. If sorbitol was unevenly mixed or temperatures differed within the vials, it became apparent when the blue dye was added, as the dye either floated near the top or sank and remained at the bottom of the vial. Where this occurred, the measurement was recorded as inconclusive. For best results, blue dye should be evenly mixed in the incubation array and sorbitol should be evenly mixed in both arrays.

Once the incubation solutions were dyed, a 1 mL syringe with a 20 Ga blunt tip needle was used to move one drop of each dyed incubation solution into the centre of the corresponding test solution cuvette of the same initial molality within the test array **[see****Supporting Information—Fig. S3****]**. Once the drop was released into the test solution, the lid was replaced quickly on the cuvette. Observations on droplet movement were made for the next 10 s; the path of a droplet was recorded: rose quickly (reached the surface within <1 s), rose slowly (did not reach the surface or took >1 s to do so), hovered (did not rise or sink after 10 s), sank quickly (reached the bottom within <1 s) or sank slowly (did not reach the bottom or took >1 s to do so). If the droplet hovered without change over 10 s, we declared that osmotic solution was a match for the TWP within the algal cells (Fig. 2, **Supporting Information—Fig. S4**). Alternatively, where no perfect ‘float’ occurred, we interpolated between which two solutions the transition from droplet rising to falling occurred.

See **Supporting Information—2.2** for methodology adjustments for use in the field.

### Laboratory experimental set up for simulated SGD and temperature

#### Algal collection

Replicate plants were collected from Sandy Beach Park (21°17ʹ12.9″N, 157°40ʹ06.6″W) at low tide and transported to the laboratory where they were kept in aerated unfiltered seawater in 0.5-gallon aquaria for 8 days to draw down tissue nutrients and acclimate to irradiances in a marine greenhouse. After the drawdown period, samples were trimmed to 0.28–0.30 g, and randomly assigned to treatment conditions in the shaded (~60 % of full sun), air-conditioned greenhouse where they remained in treatment conditions for 8 days (draw-down, pre-treatment and experiment times following Dailer *et al.* 2012).

#### Simulated treatments

Eight aerated jars were nested within temperature-controlled, water-filled bins. Treatments consisted of one simulation of non-SGD-influenced nearshore reef conditions (35 ppt and 14 µmol NO_3_^−^) and three simulated conditions of increasing SGD-influenced salinity and nutrient conditions (28 ppt and 27 µmol NO_3_, 18 ppt and 53 µmol NO_3_, 11 ppt and 80 µmol NO_3_; following Johnson *et al.* 2008; Amato *et al.* 2018). Simulation solutions were pre-mixed in bulk (~20 L) from filtered sea water and deionized water. Nitrate and phosphate standards were added inoculated into each jar. A nested study design additionally allowed for two temperature treatments (18 and 27 °C). Individual algae samples were inserted into treatment jars with 700 mL of the appropriate treatment solution. Every 2 days, water and nutrient combinations were replenished for each respective algal sample and jar locations were shifted within the bins to minimize position effects. On the morning of day 9, TWP was measured using the Chardakov methodology, following growth and photosynthesis determinations (not shown).

### Chardakov methodology standard measurements

#### Solution temperature

To investigate whether temperature affects TWP, temperature was measured in incubation and test solutions. Although efforts were made to maintain constant temperatures within the Chardakov arrays, the data revealed that temperatures within the arrays varied even while in an air-conditioned room (22 °C). The mean difference between the incubation array and the corresponding test array was 1.2 °C ± 1.5. We determined that a temperature difference of <1 °C was ideal, but a difference of <3 °C was acceptable, as inconclusive analyses did not occur in this range. Inconclusive analyses sometimes occurred outside this range, where droplets did not rise or sink in a sequential fashion and no ‘hover’ was seen. See **Supporting Information—2.3** for additional discussion.

#### Incubation times

To examine the potential effects of variable incubation times, we recorded the time that each sample was entered into the incubation vials, and the time that the Chardakov analysis occurred. From this, we calculated the incubation time for each sample and examined any effects of incubation time on measured TWP. In order to standardize incubation time, it is critical to stagger insertion of samples into the incubation vials to allow time for analysis. Having additional users, one to insert fragments and one or more to analyse results, is preferred.

### Statistical methods

To determine which factors affect TWP in our laboratory experiment, we created linear models using the lm function within the stats package in R (R Core Team 2022). Fixed predictors for the final model included species, simulated salinity/nutrient treatments, temperature treatments, incubation time and temperature differences between incubation and test solutions. We also included a block to account for the date of analysis. Preliminary models found no interactions between the predictors, so interactions were removed from the final model. We then conducted a type two analysis of variance with an alpha level of 0.05 using the ANOVA function within the car package in R (Fox and Weisberg 2018; R Core Team 2022).

## Results

### Tissue water potential for *H. musciformis* and *U. lactuca* under simulated SGD conditions

**Figure 4.**
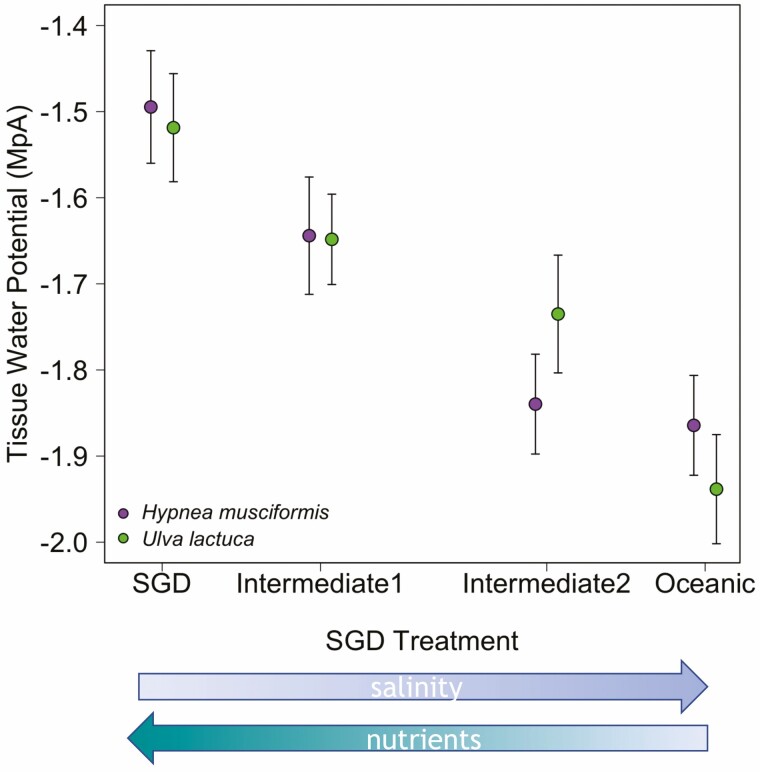
Tissue water potential (TWP) means with standard error for TWP for each species after only 8 days under each salinity/nutrient treatment. Simulated treatments are shown along the x axis from ambient nearshore marine conditions to increasingly SGD-influenced nearshore reef conditions.

Both species demonstrated a significant positive correlation between TWP and SGD salinity/nutrient treatment (Fig. 4). The final linear model included all main effects and found a significant relationship between TWP and SGD treatment (*F*_1_ = 59.79, *P* < 0.0001), and found no significant relationship with temperature treatment (*P* = 0.94), species (*P* = 0.59), incubation time (*P* = 0.39) or temperature differences (*P* = 0.96). The block for date showed no significance between TWP and analysis date.

## Discussion

The Chardakov method is a reliable, simple and cost-effective method for measuring macroalgal TWP. Here, we demonstrated its applicability for two algal species with different morphologies. We identified a significant positive relationship between TWP and simulated SGD-influence for both *U. lactuca* and *H. musciformis*, reflecting a change in TWP for both species after 8 days in response to external osmotic conditions and nutrient availability. These results reflect the ecologically expected response in TWP to a gradient of fully saline, oligotrophic marine conditions to SGD-influenced nutrient-enriched and hypo-osmotic conditions. Tissue water potential was more negative under full saline conditions and less negative under low salinity, high nitrogen SGD conditions. Unexpectedly, both species demonstrated a similar response to TWP. These results suggest that the TWP values needed to survive under SGD conditions may be constrained, and address gaps in understanding of algal physiological ecology in SGD-influenced systems (Dulai *et al.* 2021).

Further examination of growth and photosynthesis in combination with TWP may reveal species-specific costs to maintaining TWP under SGD conditions, as the pathways and organic osmolytes involved in TWP regulation vary across taxonomies (Kirst 1989). Respiratory costs for maintenance of TWP at osmotic extremes have not yet been examined. Nevertheless, the TWP values measured here are within the range of values previously measured for macroalgae (Kirst 1977), and demonstrate an 8-day response time for both species in the ability to adjust to osmotic challenges. Further, we provide an updated Chardakov methodology and approach, which improves the efficiency and accuracy in taking measures of TWP. To date, this approach has been used with tropical and sub-tropical species: *H. musciformis and U. lactuca*, as well as additional Rhodophytes: *Acanthophora spicifera*, *Gracilaria salicornia*, *Hydropuntia perplexa*, *Laurencia dendroidea* and *Laurencia mcdermidae*.

Significant knowledge gaps remain regarding TWP response that could be addressed by this methodology, including: other SGD-associated species, across other gradients including estuaries and tide pools, relationships to salinity tolerance in selection of ecotypes and even definition of subspecies, and completion of life histories (Kirst 1989; Dulai *et al.* 2021). Osmoregulation may provide ecological advantages, impact distribution and be related to seasonal gamete production (Kirst 1989; Dulai *et al.* 2021). Further experimentation is needed to understand the complex interrelations between osmotic response and environmental characteristics such as salinity, temperature, nutrients, light availability, as well as with other aspects of macroalgal physiology including photosynthesis, respiration and growth. Further analyses will examine the interrelatedness between these measured physiological responses to better understand the integrated physiological response to SGD conditions by these two species.

Use of this methodology will benefit from further calibration through comparisons of Chardakov measurements to nanoreceptor or psychrometer measurements for macroalgae, similar to the work done for terrestrial plants by Knipling and Kramer (1967). Still further calibration should examine any effect of fragment size, fragment area or weight in relation to TWP measured value. Similarly, future calibration work should focus on any effects of morphology on TWP measurements and examine any variation in the time to steady-state equilibrium with sorbitol solutions for different species.

While nanoreceptors for examining water relations (Jain *et al.* 2021) represent a significant advancement that bolsters our understanding of osmoregulation, we invite the use of the Chardakov method by users of diverse economic and training levels to address knowledge gaps associated with osmotic response and examine the diverse systems, which experience salinity changes worldwide. The historical use of this methodology in teaching courses exemplifies the way that this methodology can be used to implement student-led data collection with proper training, as use of this technique is a straightforward way to collect TWP data for macroalgae in any ecological setting.

## Conclusions

The use of this updated Chardakov method addresses broad remaining knowledge gaps regarding macroalgal osmoregulation. Here, we provide a starting point to investigate osmotic responses in macroalgae using the Chardakov method across ecosystems and species, and to better understand macroalgal physiological ecology in SGD-influenced and other dynamic systems. Filling the knowledge gaps regarding macroalgal osmotic response is pivotal given global increases in anthropogenic impacts and climate change on nearshore reefs (Dulai *et al.* 2021). The consequences of anthropogenic alteration of SGD quality and quantity through groundwater pumping, pollution and alteration of groundwater flow, in combination with the effects of changing precipitation patterns, sea level rise, ocean warming and acidification are likely to influence nearshore reef dynamics and macroalgal osmotic response in complex ways (Richardson *et al.* 2017; Valle *et al.* 2019; Dulai *et al.* 2021). Even macroalgae that are tolerant to hypo-osmotic or hyper-osmotic changes have species-specific tolerance ranges, which are yet undefined for the majority of native Hawaiian macroalgae (Dulai *et al.* 2021). This work highlights the importance of experimental studies that examine osmotic responses to better project natural cycles, as well as anthropogenic and climate change impacts on macroalgae and reef ecology.

## Supporting Information

The following additional information is available in the online version of this article –

Supporting Information 1: FIGURES

**Figure S1**: Fragments of *Ulva lactuca* in an incubation array. Fragments are of similar size, color, and morphology.

**Figure S2:** Following incubation, the incubation solution is dyed using Aniline Blue dye.

**Figure S3:** A droplet of each dyed incubation solution is inserted into the centre of the vial of the test solution of the same initial molar concentration using a syringe with wide-tip.

**Figure S4:** When a droplet hovers with little to no float or sink, a match is found between the molar concentration of the incubation solution and the TWP of the plant material. In this figure, the match is 0.65 M.

**Supporting Information 2: BEST PRACTICES**

**Supporting Information 3: R CODE**

**Supporting Information 4: DATA**

plad055_suppl_Supplementary_Material

## Data Availability

The final R code used for statistical analyses in this study can be found in **Supporting Information—3**. The raw data used in these analyses can be found in **Supporting Information—4**. Additional information is available upon request from the corresponding author.

## Notes

**Form and Function. Chief Editor:** Kate McCulloh
