# Supplementary material for: Measuring tissue water potential in marine macroalgae via an updated Chardakov method
Source: AoB Plants. 2023 Aug 22;15(5):plad055. doi: 10.1093/aobpla/plad055 (PMC10601392; doi:10.1093/aobpla/plad055)
Supplement: plad055_suppl_Supplementary_Material [file plad055_suppl_supplementary_material.pdf]

SUPPORTING INFORMATION 1: FIGURES

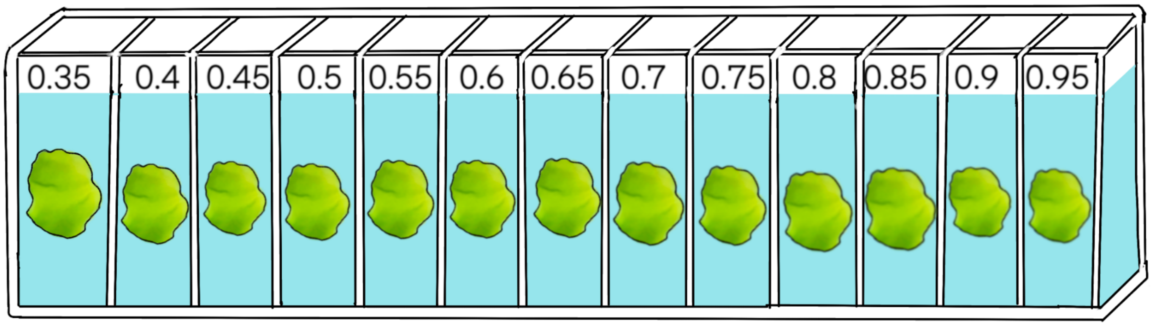

SI Figure 1: Fragments of *Ulva lactuca* in an incubation array. Fragments are of similar size, color, and morphology.

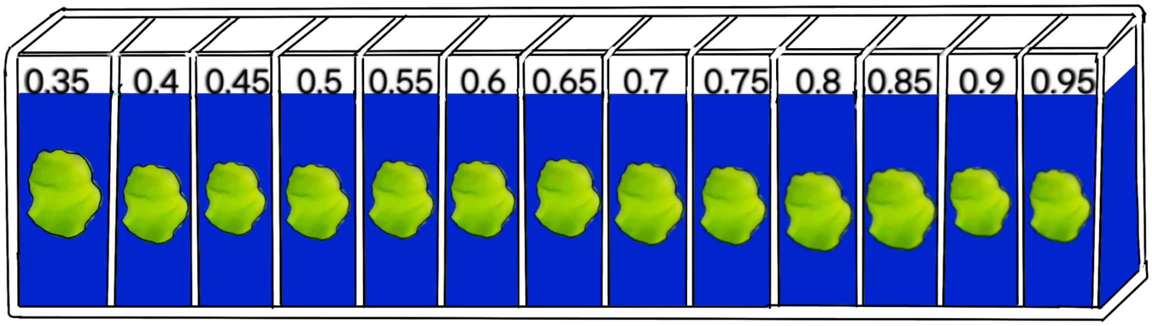

SI Figure 2: Following incubation, the incubation solution is dyed using Aniline Blue dye.

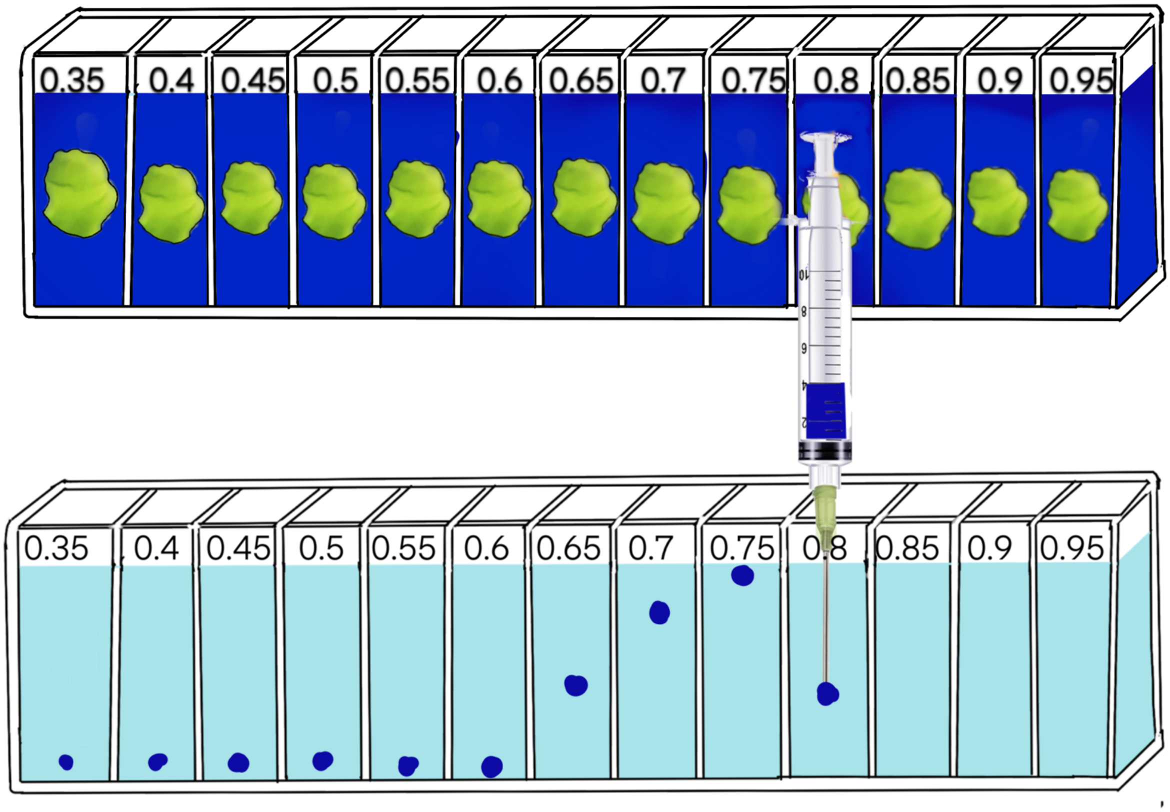

SI Figure 3: A droplet of each dyed incubation solution is inserted into the center of the vial of the test solution of the same initial molar concentration using a syringe with wide-tip.

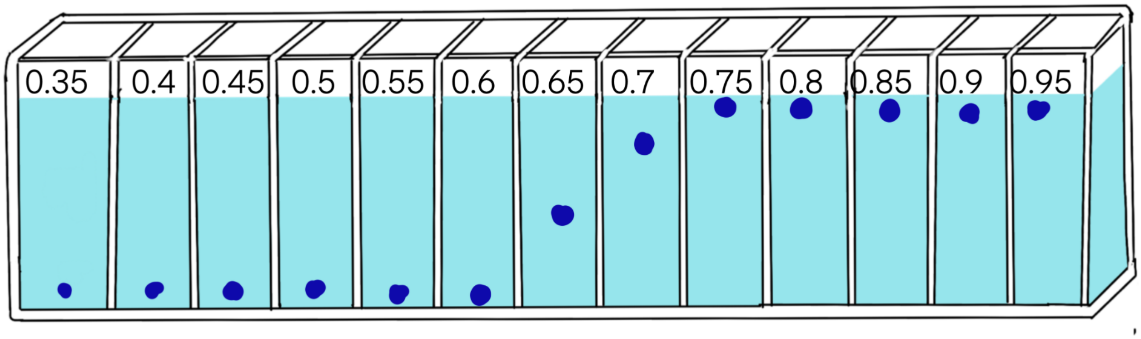

SI Figure 4: When a droplet hovers with little to no float or sink, a match is found between the molar concentration of the incubation solution and the TWP of the plant material. In this figure, the match is 0.65 M.

SUPPORTING INFORMATION 2: BEST PRACTICES

SI 2.1. Conversion of molar sorbitol values to MPa

Once measurements have been taken, the user will be left with The molar concentrations corresponding to the TWP of the tissue may. These must then be converted from the molar concentration of sorbitol to the corresponding TWP within the tissue using the water potential relationship (eq. 1). Because standard lab conditions of gravity and pressure in these experiments are constant Ψ_p and Ψ_g are both zero, yielding:

$$\Psi_w = \Psi_s \qquad \text{eq. 2}$$

which can be calculated using the following:

$$\Psi_s = -icRK \qquad \text{eq. 3}$$

where i is the ionization constant (one for sorbitol), c is molarity, R is the Universal Gas Constant, and K is the temperature in Degree Kelvin (Taiz et al. 2014).

SI 2.2 Methodology adjustments for use in the field

In addition to the laboratory experiments described here, many of the best practices for use of the Chardakov method discussed in this chapter were revealed through trial and error during fieldwork trials of this application at Waiʻalae ʻIki Beach during four field seasons, following preliminary laboratory trials. Thus, we include field methods we found most effective in this section.

For field implementation arrays were stored in plastic boxes on ice in a cooler until 1 hour leading up to measurements, when the boxes were moved to a shady and cool area under the pop-up tent used for shelter for field analyses. It is key to maintain similar temperatures within the incubation and test arrays. During field analyses, a shaker table was not used, rather each array was shaken prior to insertion of fragments or use as a test array. Avoid wind during insertion of the incubation droplets into the test solution, and quickly replace the cuvette caps after droplet insertion.

SI 2.3 Inconclusive Analyses

Inconclusive analyses occur where there is no linear change between floating and sinking of incubation droplets, and no “hover” is seen. For instance, all droplets float, or droplets nonsensically switch between floating and sinking. The most likely causes of inconclusive analyses are user error in filling vials with incorrect sorbitol solution, algal fragments added with seawater remaining on them, or too great of a temperature difference between incubation and test arrays. Similar handling of incubation arrays

and test arrays is critical to minimizing temperature differences. Each test array should be handled alongside the incubation array, including shaking and storage during incubation.

SUPPORTING INFORMATION 3: R CODE

```
library("stats")
library("vegan")
library("effects")
library("stats")
library("ggplot2")
library("dplyr")
library(car)
library(lme4)
library(lmerTest)
library(effects)

#Lanai data
lab=read.csv("lanai_data.csv")
lab$Date <- as.Date(lab$Date, "%m/%d/%y")
lab$Replicate <- as.factor(lab$Date)
plot(lab$Treatment.PPT, lab$MPA)

lab$MPA2 <- -1*(lab$MPA)
# plot(lab$Temperature, lab$MPA)
model=lm(MPA2~Treatment.PPT.*Temperature*Species + Replicate, data=lab)
plot(allEffects(model))
plot(model)
summary(model)
Anova(model, type='2')
#no three way or two way interactions

model2=lm(MPA2~Treatment.PPT. + Temperature + Species + Replicate, data=lab)
plot(allEffects(model2))
Anova(model2, type='2')

# # Response: MPA
                # Sum Sq  Df F value   Pr(>F)
# Treatment.PPT.  3.4899   1 47.4282 1.49e-10 ***
# Temperature     0.0022   1  0.0302   0.8624
# Species         0.0001   1  0.0007   0.9788
# Replicate       0.4847   4  1.6466   0.1655
# Residuals      10.9639 149

#Working temperature differences in incubation

table(lab$Algae.Temp)

table(lab$Test.Temp)

plot(lab$Algae.Temp ~ lab$Test.Temp)

lab$Temp.diff1<-lab$Algae.Temp-lab$Test.Temp
hist(lab$Temp.diff1)
table(lab$Temp.diff1)

lab$Temp.diff2<-lab$Test.Temp-lab$Initial.Temp.in.chardakov.vials
table(lab$Temp.diff2)

#making incubation time values

lab$Time1<-paste(lab$Date, lab$Time.to.incubation)
lab$Time1<-strptime(lab$Time1, format="%Y-%m-%d %H:%M:%S")

lab$Time2<-paste(lab$Date, lab$Time.in.Chardakov.vials)
lab$Time2<-strptime(lab$Time2, format="%Y-%m-%d %H:%M:%S")

lab$Time3<-paste(lab$Date, lab$Analyzed.Time)
lab$Time3<-strptime(lab$Time3, format="%Y-%m-%d %H:%M:%S")
```

```
#time between end of experiment and start Chardakov
lab$sittime1<-difftime(lab$Time2, lab$Time1)

#time in Chardakov incubation
lab$sittime2<-difftime(lab$Time3, lab$Time2)
hist(as.numeric(lab$sittime2))
#Total time
lab$sittime3<-difftime(lab$Time3, lab$Time1)

# lab$sittime1<-as.factor(lab$sittime1)
# lab$sittime2<-as.factor(lab$sittime2)
# lab$sittime3<-as.factor(lab$sittime3)

hist(lab$sittime)

lab$sittime2 <- as.numeric(lab$sittime2)

#does incubation time affect MPA?

model3=lm(MPA2~Treatment.PPT. + Temperature + Species + sittime2 + Temp.diff1 + Replicate,
data=lab)
plot(allEffects(model3))
Anova(model3, type = '2')
Response: MPA2
#               Sum Sq  Df F value    Pr(>F)
# Treatment.PPT. 4.2244   1 59.7865 6.427e-12 ***
# Temperature    0.0003   1  0.0044    0.9472
# Species        0.0206   1  0.2918    0.5902
# sittime2       0.0527   1  0.7459    0.3897
# Temp.diff1     0.0001   1  0.0019    0.9656
# Replicate      0.1365   3  0.6441    0.5883
# Residuals      7.4897 106

plot(lab$Temp.diff1 ~ lab$sittime2)

model4=lm(MPA2~Treatment.PPT.*Species + Temperature  + sittime2 + Temp.diff1 + Replicate,
data=lab)
plot(effect('Treatment.PPT.*Species', model4))

library(doBy)

meanMPA <- summaryBy(MPA2 ~ Treatment.PPT. + Species, data = lab, FUN = c(mean, sd,
length))
meanMPA$se <- meanMPA$MPA2.sd/sqrt(meanMPA$MPA2.length)

library(gplots)



par(mar = c(6,6.5,2,2), mgp = c(4.5, 1, 0), lwd = 1.5)
plotCI( y = meanMPA$MPA2.mean[which(meanMPA$Species == 'Hypnea')], x =
meanMPA$Treatment.PPT[which(meanMPA$Species == 'Hypnea')],
        uiw = meanMPA$se[which(meanMPA$Species == 'Hypnea')], sfrac = 0.005, gap = 0, cex
= 2, pch = 21, pt.bg = 'purple',
        ylab = 'Tissue Water Potential (MPa)', xlab = 'Treatment', xaxt = 'n', cex.axis =
2, cex.lab  = 2, ylim = c(-2, -1.4), las = 1, xlim = c(10, 36))

axis(side = 1, at = c(11,18,28,35) + 0.5, labels = c('35ppt', '28ppt', '18ppt', '11ppt'),
cex.axis = 2)

plotCI( y = meanMPA$MPA2.mean[which(meanMPA$Species == 'Ulva')], x =
meanMPA$Treatment.PPT[which(meanMPA$Species == 'Ulva')]+1,
        uiw = meanMPA$se[which(meanMPA$Species == 'Ulva')], sfrac = 0.005, gap = 0, cex =
2, pch = 21, pt.bg = 'green',
        ylim = c(-2, -1.4), las = 1, add = T)
```

```
legend('bottomleft', pch = 21, pt.bg = c('purple','green'), legend =
c(expression(italic('Hypnea musciformis'), italic('Ulva lactuca'))), cex = 1.5, bty = 'n')


#Reverse X axis
meanMPA$Treatment.PPT.<- -(meanMPA$Treatment.PPT.)
par(mar = c(6,6.5,2,2), mgp = c(4.5, 1, 0), lwd = 1.5)
plotCI( y = meanMPA$MPA2.mean[which(meanMPA$Species == 'Hypnea')], x =
meanMPA$Treatment.PPT[which(meanMPA$Species == 'Hypnea')],
        uiw = meanMPA$se[which(meanMPA$Species == 'Hypnea')], sfrac = 0.005, gap = 0, cex
= 2, pch = 21, pt.bg = 'purple', ylab = 'Tissue Water Potential (MPa)',
        xlab = 'Treatment', xaxt = 'n', cex.axis = 2, cex.lab  = 2, ylim = c(-2, -1.4),
las = 1, xlim = c(-36, -10))

axis(side = 1, at = c(-35,-28,-18,-11) + 0.5, labels = c('35ppt', '28ppt', '18ppt',
'11ppt'), cex.axis = 2)

plotCI( y = meanMPA$MPA2.mean[which(meanMPA$Species == 'Ulva')], x =
meanMPA$Treatment.PPT[which(meanMPA$Species == 'Ulva')]+1,
        uiw = meanMPA$se[which(meanMPA$Species == 'Ulva')], sfrac = 0.005, gap = 0, cex =
2, pch = 21, pt.bg = 'green',  ylim = c(-2, -1.4), las = 1, add = T)

legend('bottomright', pch = 21, pt.bg = c('purple','green'), legend =
c(expression(italic('Hypnea musciformis'), italic('Ulva lactuca'))), cex = 1.5, bty = 'n')
```

SUPPORTING INFORMATION 4: DATA

Lanai_data

Sample#	Species	Date	Salinity (PPT)	Temp C	Result	MPA	Algae Temp	Test Temp	Analyzed Time	Time to incubation	Time in Chardakov vials	Initial Temp	Side
H41	Hypnea	10/08/21	35	27	0.45	1.111192425			09:43:00	08:22:00	08:51:00	24.9	Diamond head
H42	Hypnea	10/08/21	28	27	0.6	1.4815899			09:58:00	08:26:00	08:56:00	24.9	Diamond head
H43	Hypnea	10/08/21	18	27	0.55	1.358124075			10:10:00	08:30:00	09:05:00	24.5	Diamond head
H44	Hypnea	10/08/21	11	27	0.45	1.111192425			10:15:00	08:37:00	09:10:00	24.9	Diamond head
U41	Ulva	10/08/21	35	27	0.65	1.605055725			10:26:00	08:40:00	09:19:00	25.3	Diamond head
U42	Ulva	10/08/21	28	27	0.55	1.358124075			10:35:00	08:45:00	09:25:00	25	Diamond head
U43	Ulva	10/08/21	18	27	0.55	1.358124075			10:40:00	08:50:00	09:41:00	25.1	Diamond head
U44	Ulva	10/08/21	11	27	0.6	1.4815899			10:50:00	08:58:00	09:50:00	25.2	Diamond head
H51	Hypnea	10/08/21	35	18	0.65	1.605055725			11:02:00	08:59:00	10:00:00		Diamond head
H52	Hypnea	10/08/21	28	18	0.7	1.72852155			11:08:00	09:05:00	10:15:00		Diamond head
H53	Hypnea	10/08/21	18	18	0.6	1.4815899			11:20:00	09:12:00	10:47:00		Diamond head
H54	Hypnea	10/08/21	11	18	0.6	1.4815899			11:31:00	09:13:00	11:06:00		Diamond head
U51	Ulva	10/08/21	35	18	0.6	1.4815899			12:15:00	09:17:00	11:16:00		Diamond head
U52	Ulva	10/08/21	28	18	0.65	1.605055725			12:20:00	09:27:00	11:30:00		Diamond head
U53	Ulva	10/08/21	18	18	0.55	1.358124075			12:30:00	09:28:00	11:45:00		Diamond head
U54	Ulva	10/08/21	11	18	0.6	1.4815899			12:00:00	09:33:00	11:56:00		Diamond head
H31	Hypnea	10/08/21	35	27	0.75	1.851987375			13:50:00	11:40:00	12:43:00		Diamond head
H32	Hypnea	10/08/21	28	27	0.7	1.72852155			13:55:00	11:46:00	12:51:00		Diamond head
H33	Hypnea	10/08/21	18	27	0.8	1.9754532			14:05:00	11:45:00	12:59:00		Diamond head
H34	Hypnea	10/08/21	11	27	0.65	1.605055725			14:12:00	11:45:00	13:05:00		Diamond head
U31	Ulva	10/08/21	35	27	0.6	1.4815899			14:18:00	11:43:00	13:11:00		Diamond head
U32	Ulva	10/08/21	28	27	0.85	2.098919025			14:24:00	11:49:00	13:17:00		Diamond head
U33	Ulva	10/08/21	18	27	0.7	1.72852155			14:33:00	11:55:00	13:22:00		Diamond head
U34	Ulva	10/08/21	11	27	0.8	1.9754532			14:38:00	12:00:00	13:29:00		Diamond head
H64	Hypnea	10/08/21	35	18	0.75	1.851987375			15:59:00	09:52:00			Diamond head
H61	Hypnea	10/08/21	28	18	0.9	2.22238485			16:04:00	09:38:00			Diamond head
U62	Ulva	10/08/21	18	18	0.8	1.9754532			16:16:00	10:04:00			Diamond head
U64	Ulva	10/08/21	11	18	0.65	1.605055725			16:27:00	10:16:00			Diamond head
U61	Ulva	10/08/21	35	18	0.8	1.9754532			16:32:00	09:58:00			Diamond head
H41	Hypnea	10/09/21	35	27	0.65	1.605055725	9	12	09:37:00	08:16:00	08:36:00	6	Ewa
H42	Hypnea	10/09/21	28	27	0.8	1.9754532	15	12	09:42:00	08:18:00	08:40:00	0	Ewa
H43	Hypnea	10/09/21	18	27	0.65	1.605055725	14	12	09:49:00	08:21:00	08:51:00	9.5	Ewa
H44	Hypnea	10/09/21	11	27	0.65	1.605055725	12	14	10:01:00	08:26:00	08:56:00	12	Ewa
U41	Ulva	10/09/21	35	27	0.85	2.098919025	16	16	10:12:00	08:32:00	09:04:00	12	Ewa
U42	Ulva	10/09/21	28	27	0.55	1.358124075	1.5	16	10:18:00	08:38:00	09:04:00	12	Ewa
U43	Ulva	10/09/21	18	27	0.75	1.851987375	15	15	10:25:00	08:44:00	09:16:00	12	Ewa
U44	Ulva	10/09/21	11	27	0.7	1.72852155	12		10:30:00	08:50:00	09:20:00	12	Ewa
H11	Hypnea	10/09/21	35	18	0.8	1.9754532	19	17	11:26:00	10:06:00	10:40:00	19	Ewa
H12	Hypnea	10/09/21	28	18	0.55	1.358124075	18		11:44:00	10:10:00	10:47:00	17	Ewa
H13	Hypnea	10/09/21	18	18	0.7	1.72852155	18		11:50:00	10:15:00	10:54:00	18	Ewa
H14	Hypnea	10/09/21	11	18	0.65	1.605055725	19	17	11:26:00	10:19:00	10:32:00	16.5	Ewa
U11	Ulva	10/09/21	35	18	0.7	1.72852155	18	16	12:02:00	10:24:00	10:59:00	18	Ewa
U12	Ulva	10/09/21	28	18	0.85	2.098919025	19	19	12:21:00	10:28:00	11:04:00	18.5	Ewa
U13	Ulva	10/09/21	18	18	0.7	1.72852155	18	13	12:28:00	10:33:00	11:08:00	19.5	Ewa

U14	Ulva	10/09/21	11	18	0.6	1.4815899	18	15	12:36:00	10:36:00	11:13:00	14	Ewa
H21	Hypnea	10/09/21	35	18	0.9	2.22238485	18	17	12:57:00	10:41:00	11:31:00	13.5	Ewa
H22	Hypnea	10/09/21	28	18	0.7	1.72852155	17	12	13:08:00	10:46:00	11:41:00	12	Ewa
H23	Hypnea	10/09/21	18	18	0.6	1.4815899	18	16	13:15:00	10:51:00	11:56:00	16	Ewa
H24	Hypnea	10/09/21	11	18	0.6	1.4815899	18	17	13:24:00	10:56:00	12:05:00	18	Ewa
U21	Ulva	10/09/21	35	18	0.9	2.22238485	19	17	13:34:00	11:01:00	12:23:00	17	Ewa
U22	Ulva	10/09/21	28	18	0.85	2.098919025	18	18	13:41:00	11:06:00	12:30:00	17	Ewa
U23	Ulva	10/09/21	18	18	0.8	1.9754532	18		13:50:00	11:11:00	12:37:00	16.5	Ewa
U24	Ulva	10/09/21	11	18	0.6	1.4815899	19	17	13:54:00	11:15:00	12:46:00	16	Ewa
H31	Hypnea	10/09/21	35	27	0.75	1.851987375	18	16	14:09:00	11:20:00	12:56:00	17.5	Ewa
H32	Hypnea	10/09/21	28	27	0.85	2.098919025	18	13	14:13:00	11:25:00	13:04:00	18	Ewa
H33	Hypnea	10/09/21	18	27	0.7	1.72852155	18		14:17:00	11:31:00	13:18:00	18.5	Ewa
H34	Hypnea	10/09/21	11	27	0.65	1.605055725	18		14:23:00	11:36:00	13:27:00	19	Ewa
U31	Ulva	10/09/21	35	27	0.8	1.9754532	18	17	14:31:00	11:41:00	13:35:00	17.5	Ewa
U32	Ulva	10/09/21	28	27	0.7	1.72852155	17	16	14:36:00	11:44:00	13:44:00	15	Ewa
U33	Ulva	10/09/21	18	27	0.65	1.605055725	17	16	14:48:00	11:48:00	13:50:00	14.5	Ewa
U34	Ulva	10/09/21	11	27	0.65	1.605055725	18	17	14:54:00	11:53:00	14:01:00	14.5	Ewa
H41	Hypnea	10/19/21	35	27	0.75	1.851987375	15	15	09:40:00	08:10:00	08:43:00	8	Diamond head
H42	Hypnea	10/19/21	28	27	0.65	1.605055725	15		09:50:00	08:12:00	08:50:00	9.5	Diamond head
H43	Hypnea	10/19/21	18	27	0.55	1.358124075	14		10:00:00	08:15:00	08:55:00	8.5	Diamond head
H44	Hypnea	10/19/21	11	27	0.75	1.851987375	16	16	10:10:00	08:16:00	08:59:00	8.5	Diamond head
U41	Ulva	10/19/21	35	27	0.7	1.72852155	16		11:12:00	08:25:00	09:04:00	11.5	Diamond head
U42	Ulva	10/19/21	28	27	0.55	1.358124075	17	16	11:19:00	08:30:00	09:09:00	14	Diamond head
U43	Ulva	10/19/21	18	27	0.7	1.72852155			11:25:00	08:34:00	09:14:00	11	Diamond head
U44	Ulva	10/19/21	11	27	0.7	1.72852155			11:30:00	08:39:00	09:19:00	10	Diamond head
H11	Hypnea	10/19/21	35	18	0.65	1.605055725	18		12:28:00	09:53:00	10:17:00	17.5	Diamond head
H12	Hypnea	10/19/21	28	18	0.85	2.098919025	18	18	12:21:00	09:59:00	10:34:00	17	Diamond head
H13	Hypnea	10/19/21	18	18	0.8	1.9754532	18		11:50:00	10:04:00	10:50:00	16.5	Diamond head
H14	Hypnea	10/19/21	11	18	0.75	1.851987375	17	18	11:40:00	10:10:00	11:06:00	18	Diamond head
U11	Ulva	10/19/21	35	18	0.85	2.098919025	18	17	12:33:00	10:16:00	11:20:00	18	Diamond head
U12	Ulva	10/19/21	28	18	0.75	1.851987375	18	18	12:39:00	10:20:00	11:40:00	20	Diamond head
U13	Ulva	10/19/21	18	18	0.75	1.851987375	18	19	12:50:00	10:26:00	11:54:00	17	Diamond head
U14	Ulva	10/19/21	11	18	0.7	1.72852155	17	17	13:01:00	10:32:00	11:59:00	18	Diamond head
H21	Hypnea	10/19/21	35	18	0.75	1.851987375	17	17	13:10:00	10:36:00	12:04:00	16	Diamond head
H22	Hypnea	10/19/21	28	18	0.65	1.605055725	17	17	13:21:00	10:40:00	12:08:00	16	Diamond head
H23	Hypnea	10/19/21	18	18	0.45	1.111192425	17	17	13:26:00	10:46:00	12:13:00	16.5	Diamond head
H24	Hypnea	10/19/21	11	18	0.5	1.23465825	18	17	13:34:00	10:52:00	12:20:00	15	Diamond head
U21	Ulva	10/19/21	35	18	0.7	1.72852155	18	18	13:52:00	11:03:00	12:28:00	15.5	Diamond head
U22	Ulva	10/19/21	28	18	0.75	1.851987375	17	18	13:55:00	11:03:00	12:36:00	16	Diamond head
U23	Ulva	10/19/21	18	18	0.75	1.851987375	17	18	14:05:00	11:08:00	12:43:00	18	Diamond head
U24	Ulva	10/19/21	11	18	0.5	1.23465825	19	18	14:17:00	11:16:00	12:50:00	16.5	Diamond head
H31	Hypnea	10/19/21	35	27	0.8	1.9754532	18	17	14:29:00	11:23:00	13:00:00	17.5	Diamond head
H32	Hypnea	10/19/21	28	27	0.75	1.851987375	19	19	14:43:00	11:23:00	13:12:00	18	Diamond head
H33	Hypnea	10/19/21	18	27	0.85	2.098919025	19	18	14:49:00	11:30:00	13:26:00	18.5	Diamond head
H34	Hypnea	10/19/21	11	27	0.45	1.111192425	20	19	15:03:00	11:36:00	13:38:00	19	Diamond head
U31	Ulva	10/19/21	35	27	0.7	1.72852155	16	17	15:12:00	11:40:00	13:44:00	8	Diamond head
U32	Ulva	10/19/21	28	27	0.7	1.72852155	17	16	15:31:00	11:44:00	14:00:00	6	Diamond head
U33	Ulva	10/19/21	18	27	0.55	1.358124075	15	15	15:25:00	11:48:00	14:07:00	7	Diamond head
U34	Ulva	10/19/21	11	27	0.5	1.23465825	15	15	15:31:00	11:52:00	14:15:00	8	Diamond head

H41	Hypnea	10/29/21	35	27	0.7	1.72852155	15	12	10:00:00	08:15:00	09:08:00	-1	Diamond head
H42	Hypnea	10/29/21	28	27	0.9	2.22238485	13	15	10:10:00	08:19:00	09:14:00	-1	Diamond head
H43	Hypnea	10/29/21	18	27	0.6	1.4815899	15	15	10:20:00	08:23:00	09:20:00	-1	Diamond head
H44	Hypnea	10/29/21	11	27	0.55	1.358124075	14	14	10:30:00	08:27:00	09:37:00	-1	Ewa
U41	Ulva	10/29/21	35	27	0.9	2.22238485	16	14	11:20:00	08:31:00	09:37:00	-1	Ewa
U42	Ulva	10/29/21	28	27	0.65	1.605055725	16	16	11:30:00	08:33:00	09:42:00	-1	Ewa
U43	Ulva	10/29/21	18	27	0.6	1.4815899	17	15	11:40:00	08:39:00	09:48:00	-0.5	Ewa
U44	Ulva	10/29/21	11	27	0.5	1.23465825	18	16	11:58:00	08:43:00	09:54:00	14	Ewa
H11	Hypnea	10/29/21	35	18	0.85	2.098919025	19	18	12:07:00	09:57:00	10:24:00	18.5	Ewa
H12	Hypnea	10/29/21	28	18	0.85	2.098919025	18	17	12:12:00	10:03:00	10:33:00	13	Ewa
H13	Hypnea	10/29/21	18	18	0.75	1.851987375	17	18	12:33:00	10:09:00	10:48:00	13	Ewa
H14	Hypnea	10/29/21	11	18	0.65	1.605055725	18	18	12:41:00	10:12:00	10:59:00	13.5	Ewa
U11	Ulva	10/29/21	35	18	0.9	2.22238485	19	18	12:50:00	10:13:00	11:18:00	15	Ewa
U12	Ulva	10/29/21	28	18	0.8	1.9754532	18	18	13:04:00	10:15:00	11:26:00	15.5	Ewa
U13	Ulva	10/29/21	18	18	0.6	1.4815899	18	18	13:13:00	10:21:00	11:33:00	16.5	Ewa
U14	Ulva	10/29/21	11	18	0.4	0.9877266	19	18	13:23:00	10:27:00	11:41:00	16	Ewa
H21	Hypnea	10/29/21	35	18	0.8	1.9754532	18	18	13:33:00	10:33:00	11:54:00	17	Ewa
H22	Hypnea	10/29/21	28	18	0.7	1.72852155	19	18	13:43:00	10:39:00	12:03:00	18	Ewa
H23	Hypnea	10/29/21	18	18	0.65	1.605055725	19	18	13:48:00	10:45:00	12:19:00	17.5	Ewa
H24	Hypnea	10/29/21	11	18	0.75	1.851987375	18	19	13:53:00	10:49:00	12:27:00	18	Ewa
U21	Ulva	10/29/21	35	18	0.95	2.345850675	18	18	13:58:00	10:56:00	12:36:00	17.5	Ewa
U22	Ulva	10/29/21	28	18	0.8	1.9754532	19	14	14:05:00	11:00:00	12:47:00	18	Ewa
U23	Ulva	10/29/21	18	18	0.7	1.72852155	19	14	14:11:00	11:05:00	12:52:00	18.5	Ewa
U24	Ulva	10/29/21	11	18	0.45	1.111192425	19	15	14:19:00	11:07:00	13:00:00	19	Ewa
H31	Hypnea	10/29/21	35	27	0.8	1.9754532	20	13	14:25:00	11:14:00	13:10:00	19.5	Ewa
H32	Hypnea	10/29/21	28	27	0.85	2.098919025	19	14	14:35:00	11:18:00	13:21:00	20	Ewa
H33	Hypnea	10/29/21	18	27	0.6	1.4815899	17	13	14:43:00	11:24:00	13:31:00	13	Ewa
H34	Hypnea	10/29/21	11	27	0.4	0.9877266	17	16	14:52:00	11:29:00	13:44:00	12.5	Ewa
U31	Ulva	10/29/21	35	27	0.9	2.22238485	16	16	14:59:00	11:32:00	13:54:00	12.5	Ewa
U32	Ulva	10/29/21	28	27	0.9	2.22238485	16	15	15:10:00	11:36:00	14:02:00	12.5	Ewa
U33	Ulva	10/29/21	18	27	0.8	1.9754532	16	16	15:15:00	11:40:00	14:11:00	13.5	Ewa
U34	Ulva	10/29/21	11	27	0.75	1.851987375	18	17	15:25:00	11:46:00	14:19:00	14	Ewa
H11	Hypnea	11/12/21	35	18	0.7	1.72852155	13	9	09:29:00	08:30:00	08:58:00	11	Diamond head
H12	Hypnea	11/12/21	28	18	0.7	1.72852155	14	12	09:45:00	08:38:00	09:03:00	11	Diamond head
H13	Hypnea	11/12/21	18	18	0.65	1.605055725	17	20	10:00:00	08:43:00	09:03:00	11	Diamond head
H14	Hypnea	11/12/21	11	18	0.65	1.605055725	21	20	10:16:00	08:50:00	09:11:00	10	Diamond head
U11	Ulva	11/12/21	35	18	0.7	1.72852155	19	16	10:27:00	08:53:00	09:20:00	9	Diamond head
U12	Ulva	11/12/21	28	18	0.6	1.4815899	21	18	10:37:00	09:00:00	09:26:00	10	Diamond head
U13	Ulva	11/12/21	18	18	0.55	1.358124075	19	20	10:47:00	09:03:00	09:26:00	14	Diamond head
U14	Ulva	11/12/21	11	18	0.5	1.23465825	21	20	10:57:00	09:06:00	09:37:00	14.5	Diamond head
H21	Hypnea	11/12/21	35	18	0.85	2.098919025	19	20	11:07:00	09:10:00	10:02:00	17	Diamond head
H22	Hypnea	11/12/21	28	18	0.6	1.4815899	21	21	11:15:00	09:15:00	10:12:00	16.5	Diamond head
H23	Hypnea	11/12/21	18	18	0.5	1.23465825	23	22	12:35:00	09:21:00	10:22:00	17.5	Diamond head
H24	Hypnea	11/12/21	11	18	0.5	1.23465825	22	22	12:40:00	09:23:00	10:35:00	20.5	Diamond head
U21	Ulva	11/12/21	35	18	0.85	2.098919025	21	22	12:45:00	09:31:00	10:49:00	16	Diamond head
U22	Ulva	11/12/21	28	18	0.75	1.851987375	21	22	12:50:00	09:32:00	11:01:00	16	Diamond head
U23	Ulva	11/12/21	18	18	0.7	1.72852155	22	22	12:55:00	09:39:00	11:10:00	15	Diamond head
U24	Ulva	11/12/21	11	18	0.7	1.72852155	22	22	13:00:00	09:42:00	11:20:00	15	Diamond head
H31	Hypnea	11/12/21	35	27	0.9	2.22238485	22	22	13:05:00	09:46:00	11:28:00	15	Diamond head

H32	Hypnea	11/12/21	28	27	0.8	1.9754532	22	22	13:10:00	09:52:00	11:39:00	15	Diamond head
H33	Hypnea	11/12/21	18	27	0.9	2.22238485	22	22	13:20:00	09:54:00	11:48:00	16	Diamond head
H34	Hypnea	11/12/21	11	27	0.8	1.9754532	22	22	13:30:00	09:59:00	11:57:00	16	Diamond head
U31	Ulva	11/12/21	35	27	0.95	2.345850675	22	22	13:40:00	10:03:00	12:05:00	16	Diamond head
U32	Ulva	11/12/21	28	27	0.55	1.358124075	22	22	13:50:00	10:09:00	12:14:00	17.5	Diamond head
U33	Ulva	11/12/21	18	27	0.5	1.23465825	22	22	14:00:00	10:12:00	12:24:00	16.5	Diamond head
U34	Ulva	11/12/21	11	27	0.8	1.9754532	22	22	14:10:00	10:16:00	12:50:00	18	Diamond head
H41	Hypnea	11/12/21	35	27	0.85	2.098919025	23	23	14:20:00	10:22:00	12:58:00	18.5	Diamond head
H42	Hypnea	11/12/21	28	27	0.8	1.9754532	23	23	14:30:00	10:27:00	13:08:00	19	Diamond head
H43	Hypnea	11/12/21	18	27	0.75	1.851987375	23	23	14:40:00	10:31:00	13:15:00	19	Diamond head
H44	Hypnea	11/12/21	11	27	0.5	1.23465825	23	23	14:50:00	10:36:00	13:23:00	19	Diamond head
U41	Ulva	11/12/21	35	27	0.7	1.72852155	23	23	15:00:00	10:41:00	13:30:00	19	Diamond head
U42	Ulva	11/12/21	28	27	0.55	1.358124075	23	23	15:05:00	10:46:00	13:35:00	19	Diamond head
U43	Ulva	11/12/21	18	27	0.65	1.605055725	23	23	15:10:00	10:49:00	13:44:00	20	Diamond head
U44	Ulva	11/12/21	11	27	0.6	1.4815899	23	23	15:15:00	10:53:00	13:50:00	20	Diamond head
